# Effects of Exposure to Differentially Stressed *Pinus sylvestris* Seedlings on the Susceptibility of Receivers to Feeding by the Large Pine Weevil

**DOI:** 10.1007/s10886-026-01688-5

**Published:** 2026-02-21

**Authors:** Sara Mashhadi Meyghani, Muhammad Usman Rasheed, James D. Blande

**Affiliations:** Department of Environmental and Biological Sciences, University of Eastern Finland, P.O. Box 1627, Kuopio, 70211 Finland

**Keywords:** Damage-induced plant volatiles, Scots pine, Plant–plant interaction, Herbivore behaviour

## Abstract

Plants release volatile organic compounds (VOCs) in response to herbivory or damage as part of an indirect defence strategy. These broadly defined damage-induced plant volatiles (DIPVs), include herbivore-induced plant volatiles (HIPVs). DIPVs elicit defence-related responses in neighbouring plants, which may influence herbivore behaviour. Although DIPVs have been studied in conifers, little is known about how they influence neighbouring plants and shape subsequent interactions with herbivores. We tested whether DIPVs released by damaged Scots pine (*Pinus sylvestris*) seedlings alter the susceptibility of undamaged receivers to feeding by the large pine weevil (*Hylobius abietis*). A two-stage experiment examined the effects of exposure to VOCs from mechanically damaged, pine weevil-damaged, sawfly-damaged, and undamaged seedlings on neighbouring receivers. In the first stage, receivers were exposed to VOCs for seven days. VOC emissions from receivers were collected and analysed using gas chromatography–mass spectrometry. Analyses revealed treatment-specific volatile profiles. In the second stage, weevil orientation was tested, and receiver susceptibility evaluated by quantifying bark damage area using digital photograph analysis and classification of feeding-damage type as superficial or deep. Weevils first oriented towards the control receivers most frequently before being found in greater numbers on receivers exposed to DIPVs from mechanically damaged and weevil-damaged neighbours. Towards the end of the experiment, they were found on receivers exposed to sawfly-induced DIPVs. The control group had more deep damage than superficial damage, whereas all DIPV exposure groups had more superficial than deep feeding spots, suggesting that prior VOC exposure may result in surface-level feeding. These findings show that DIPVs can trigger changes in receivers that influence pine weevil orientation and feeding behaviour.

**Supplementary Information:**

The online version contains supplementary material available at 10.1007/s10886-026-01688-5.

## Introduction

Plants interact with a wide range of organisms, including herbivores and neighbouring plants (Mescher and De Moraes 2015; Boncan et al. 2020; Rasheed et al. 2023). One of their key communication and defence strategies involves the emission of volatile organic compounds (VOCs) (Holopainen and Blande 2012; Kessler et al. 2023). These compounds can influence herbivore host selection, i.e., the process by which herbivores choose which plants to feed on. They can also activate a range of biochemical defences that deter herbivory and limit herbivore performance (War et al. 2012; Belete 2018; Singh and Singh 2021). Among these volatiles, herbivore-induced plant volatiles (HIPVs), a major subgroup of damage-induced plant volatiles (DIPVs), are ecologically important compounds released in response to herbivore attack (Arimura et al. 2005; Venkatesan 2015; Pearse et al. 2020). HIPVs play roles in both plant defence and communication. They act as an indirect defence by attracting natural enemies of herbivores—such as predators and parasitoids—thereby reducing herbivore pressure. They also function as a direct defence by repelling herbivores and influencing their oviposition behaviour (De Moraes et al. 2001). In addition, HIPVs can prime neighbouring plants to activate their own defences by inducing defence-related gene expression and enhancing the production of secondary metabolites (Holopainen and Blande 2013; Girón-Calva et al. 2016; Gebreziher 2018; Michereff et al. 2021). This priming effect may lead to enhanced resistance, reduced herbivory, and increased emission of volatiles upon later attack (Engelberth et al. 2004; Morrell and Kessler 2014). DIPVs play crucial roles in mediating plant–plant and plant–insect interactions, contributing to plant adaptation to environmental stresses such as herbivory, pathogen attack, and abiotic factors. By understanding the mechanisms of induced resistance, we can predict which herbivores will be affected by induced responses (Mumm and Hilker 2006; Heil 2008; War et al. 2012; Pierik et al. 2014; Rosenkranz et al. 2021).

Studies have shown that plants can actively respond to HIPVs from damaged neighbours by modifying their own VOC profiles (Ninkovic et al. 2019, 2021; Yu et al. 2022; Rasheed et al. 2023), enabling them to react more quickly and effectively to upcoming biotic and abiotic damage or stress factors, such as herbivore attack and mechanical wounding (Niinemets 2010; Midzi et al. 2022). In addition to herbivory, mechanical injury and other forms of abiotic damage can also trigger the emission of DIPVs (Niinemets 2010; Midzi et al. 2022). These volatiles are released upon tissue injury and may act as early stress signals, activating defence-related pathways in undamaged parts of the same plant or in neighbouring plants (Matsui and Koeduka 2016; Engelberth 2021; Midzi et al. 2022; Mostafa et al. 2022).

Extensive research has characterized induced VOC emission as a “cry for help” that enhances direct and indirect plant defences (Dicke and Baldwin 2010; Mauch-Mani et al. 2017). It is also recognized as a mediator of plant–plant interactions that result in the priming of neighbouring plants (Dicke and Baldwin 2010; Loreto and Schnitzler 2010; Yoneya and Takabayashi 2014; Midzi et al. 2022; Rasheed et al. 2023). However, the comparative attractiveness of primed plants to herbivores remains poorly understood. DIPVs have been observed to render undamaged receiver plants both more (e.g. *Brassica oleracea* plants exposed to volatiles from conspecific neighbours damaged by *Plutella xylostella* larvae showed increased susceptibility to oviposition) (Li and Blande 2015) and less (e.g. undamaged cotton and alfalfa plants exposed to HIPVs emitted by conspecific cotton plants damaged by larvae of *Spodoptera littoralis*, which resulted in reduced oviposition on the receiver plants by *S. littoralis*) (Zakir et al. 2013) susceptible to oviposition by herbivores in herbaceous species, which makes the end result of an interaction difficult to predict.

Scots pine (*Pinus sylvestris L.*, Pinaceae), a dominant tree in boreal forests, emits large quantities of VOCs, especially monoterpenes, which are involved in both constitutive (pre-formed) and induced (stress-responsive) defences (Delorme and Lieutier 1990; Ghimire et al. 2013; Kovalchuk et al. 2015). Exposure to HIPVs released by damaged Scots pine has been shown to prime neighbouring seedlings for a stronger and more rapid response upon subsequent herbivory and to increase their resistance to herbivore damage (Yu et al. 2022, 2024). The large pine weevil (*Hylobius abietis;* Coleoptera: Curculionidae) is among the most destructive pests affecting Scots pine regeneration, particularly in newly established plantations adjacent to recently clear-cut areas, where seedlings face a high risk of bark feeding that can result in girdling and subsequent mortality (Örlander et al. 2000). Feeding by pine weevils induces increased emissions of VOCs both locally from the bark and systemically from the needles, leading to significant changes in the plant’s overall volatile profile (Heijari et al. 2011; Kovalchuk et al. 2015). Importantly, exposure to HIPVs from pine weevil-damaged conspecifics has been shown to reduce subsequent bark damage in nearby seedlings (Yu et al. 2022; Rasheed et al. 2023).

The European pine sawfly (*Neodiprion sertifer;* Hymenoptera: Diprionidae) is known to defoliate Scots pine seedlings (Lyytikäinen 1992; Lyytikäinen-Saarenmaa and Tomppo 2002). Scots pine can respond to early insect cues, such as exposure to sawfly sex pheromones, which may prime its defences and reduce egg survival (Bittner et al. 2019). Feeding by sawfly larvae causes a strong local increase in volatile emissions in the attacked pine, potentially serving as a chemical signal to neighbouring plants (Ghimire et al. 2013). Although plant responses to early insect attacks, including oviposition, have been investigated (Ghimire et al. 2013; Bittner et al. 2019; Rahman Soad 2025), there is currently no clear experimental evidence showing that feeding by the European pine sawfly induces defence priming in adjacent Scots pines. Nevertheless, previous studies on other herbivores suggest that, through VOC-mediated signalling, Scots pine may enhance its own defences and potentially influence the responses of neighbouring plants (Semiz et al. 2012; Kovalchuk et al. 2015; Yu et al. 2022).

Volatile-mediated plant–plant interactions have been shown to influence plant susceptibility to herbivores and to support both direct and indirect defence mechanisms (Girón-Calva et al. 2016; Blande 2017). Such interactions influence community-level signalling, shape plant growth and survival, and help structure ecological processes across trophic levels (Ninkovic et al. 2021; Kessler et al. 2023). Understanding how plants interact with and respond to biotic and abiotic stressors remains an important ecological question, particularly under increasing environmental pressures. While volatile-mediated interactions are well documented in a variety of plant systems, including herbaceous and woody plants, such as conifers (Yu et al. 2022; Kessler et al. 2023), less is known about how prior exposure to stress-induced volatiles from damaged neighbours influences herbivore feeding behaviour on undamaged receiver seedlings. This study provides new insights into how pre-exposure to VOCs released by neighbours under different stressors—mechanical damage, pine weevil feeding, or sawfly larval feeding—affects the attractiveness of undamaged Scots pine receiver seedlings to the large pine weevil. We hypothesized that exposure to VOCs from differently stressed emitter seedlings would differentially affect the susceptibility of receiver plants to herbivore-feeding. 

## Methods and Materials

### Plant and Insect Material

A total of approximately 300 one-year-old Scots pine seedlings were purchased from the Natural Resources Institute Finland (Luke) research station in Suonenjoki, Finland. The seedlings were potted in a 1:1 mixture of sand and peat, sourced from Weber Hiekkatuote. Seedlings were maintained outdoors under natural light and temperature conditions for approximately six months. They were fertilized once a week with 0.1% N–P–K (12–5–27) fertilizer (Taimi Superex, Kekkilä Oyj, Finland). Twenty-four hours prior to the start of the experiment, the seedlings were transferred to a growth room for acclimatization to indoor conditions. Only seedlings of similar height (35–38 cm) and of the α-pinene chemotype (based on their VOC profile) were selected to reduce variation. Altogether, 104 seedlings were used across two experimental runs of 52 seedlings each. Weevils were hand-collected on 20 June 2024 from a sawmill located in Suonenjoki (62.6638636, 27.0082250). After collection, adult weevils were stored short-term at 8 °C in plastic containers in a laboratory refrigerator and supplied with fresh pine twigs as food. Refrigeration was used to reduce metabolic activity and maintain the insects in a quiescent state. Prior to experiments, weevils were transferred to room temperature and starved for 24 h to standardize hunger levels. Sawfly larvae were collected on 27 June 2024 from an outbreak area in Puumala (61.5209167, 28.0537500), Southern Savonia. The forest was predominantly composed of Scots pine, with some Norway spruce (*Picea abies*). Larvae were selected based on similar body size, which corresponded to mid instars (second or third instar) prior to the experiments. Larvae were kept in growth chambers (day: 18 h light; night: 6 h dark; temperature: 16 °C; humidity: 60%) and provided with pine twigs until use.

### Experimental Setup

The experiment was conducted at the University of Eastern Finland in two clearly separated phases. Phase 1 was designed to expose receiver seedlings to volatile organic compounds (VOCs) emitted by stressed or control neighbours, while Phase 2 assessed weevil behavioural responses in enclosed experimental cages.

#### Phase 1: VOC Exposure Experiment

**Fig. 1:**
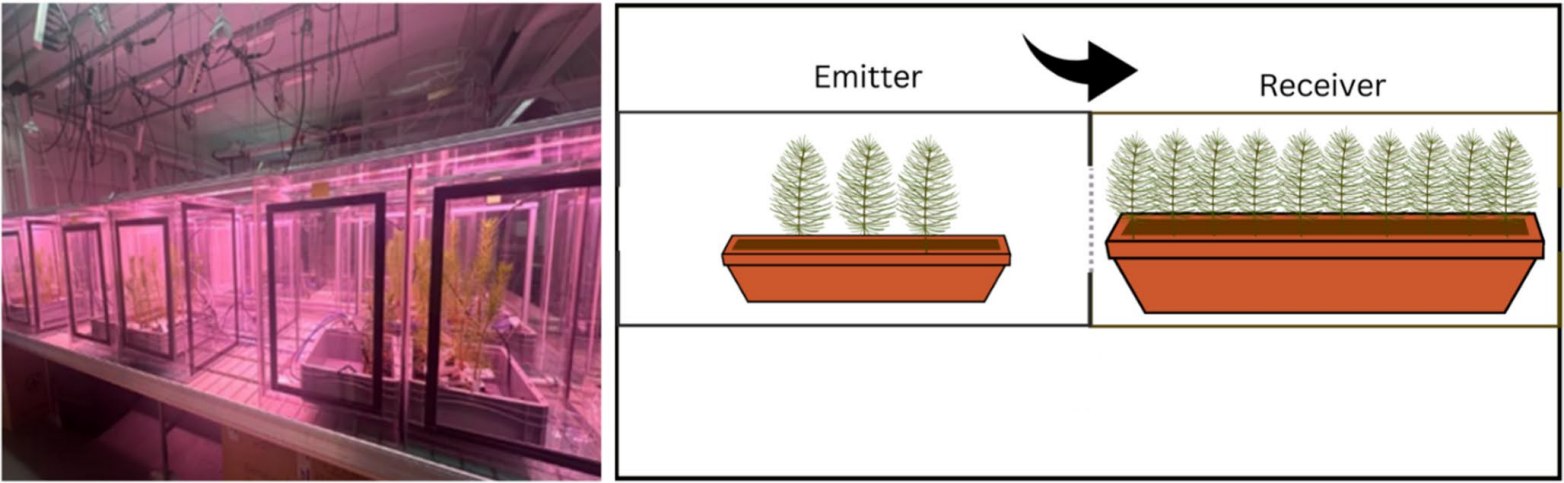
Experimental setup for plant–plant interaction study. The left panel shows the experimental chambers used in the study. The right panel shows a schematic of the emitter–receiver arrangement. Airflow was directed from the emitter chamber toward the receiver chamber (black arrow). The two chambers were separated by a shared wall containing a mesh-covered opening (grey dotted line), which allowed air exchange while preventing insect movement between chambers

On 30 June 2024, Scots pine seedlings were transferred to ventilated tabletop plastic plant growth chambers (45 × 45 × 70 cm) with detachable walls, allowing each pair of chambers to be connected side by side. Each emitter–receiver chamber pair shared a common wall containing a large rectangular opening (approximately 42 × 30 cm), which was fully covered with a fabric mesh (0.5 × 0.5 mm aperture) that allowed airflow between chambers while preventing herbivores from crossing between emitter and receiver compartments. Airflow in Phase 1 was mechanically driven and strictly unidirectional. Dry, filtered air was supplied to the emitter chamber using a dry air generator and flowed from the emitter chamber toward the receiver chamber through the mesh-covered opening. Clean air entered the emitter chamber at a flow rate of 3.9 L min⁻¹, while VOC-containing air exited the receiver chamber at 1.9 L min⁻¹. The lower outflow relative to inflow maintained a higher pressure inside the chamber system relative to the ambient laboratory, preventing ingress of external air and ensuring consistent VOC transfer from emitters to receivers. As a result, receiver seedlings were exposed exclusively to VOCs emitted by either stressed or control emitters, without direct contact or insect transfer. Each chamber pair consisted of three emitter and ten receiver seedlings, randomly selected using a random number generator from all available seedlings and assigned to emitter or receiver positions within the chambers. Seedlings placed on the left side of the chambers were designated as emitters, while those on the right side were considered receivers, arranged in two parallel rows (Fig. 1).

Environmental conditions were monitored throughout the experiment, including average air humidity (76.2 ± 7.1%), soil moisture (28.3 ± 8%), soil temperature (22.6 ± 0.5 °C), and air temperature (23.4 ± 0.7 °C). The light cycle was a 16-h light and 8-h dark photoperiod (16 L:8 D) provided by LED lamps (Valoya B100, NS1; Valoya Oy, Finland; 93 W). During the light period, photosynthetically active radiation (PAR) was maintained at approximately 250 µmol m⁻² s⁻¹.

Phase 1 was conducted twice on two consecutive days using independent sets of seedlings. In each experimental run, 52 seedlings were used (three emitters and ten receivers per chamber). These were assigned to four exposure treatments: control (C), mechanical damage (M-EXP), weevil exposure (W-EXP), and sawfly exposure (S-EXP). Control emitters received no damage treatment. In the M-EXP treatment, a narrow strip of bark (~ 0.5 cm in length) was removed daily from the stem of each emitter seedling at a comparable height using a razor blade. In the W-EXP treatment, three pine weevils were placed on each emitter seedling (nine per chamber), while in the S-EXP treatment, ten European pine sawfly larvae were placed on each emitter seedling (30 per chamber). Although weevils and sawfly larvae feed on different tissues (stem bark versus needles), all treatments were applied consistently across seedlings to induce biologically relevant stress signals. All treatments were maintained for seven days. After the exposure period, two receiver seedlings per treatment (*n* = 8 per run) were selected for VOC analysis, and the remaining receivers were used in Phase 2.

#### Phase 2: Weevil Behavioural Assay

**Fig. 2:**
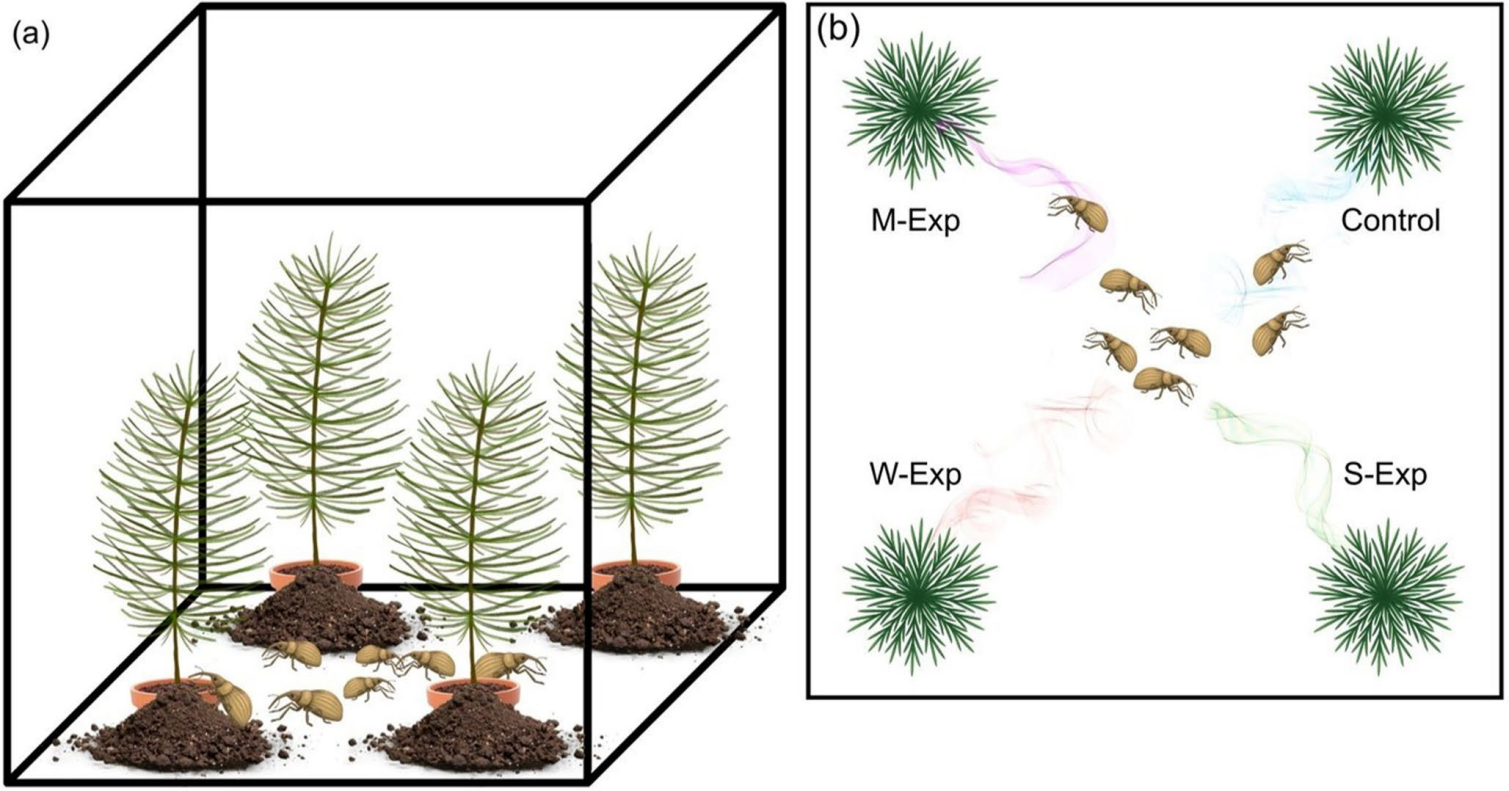
Schematic representation of one experimental cage. The left (**a**) panel illustrates a cage containing receiver seedlings exposed to volatiles from emitters subjected to four treatments: mechanical damage (M-EXP), weevil feeding (W-EXP), sawfly feeding (S-EXP), and undamaged control. Seven pine weevils were released into the centre of the cage, and seedling attractiveness was assessed from (i) weevil orientation (distribution among seedlings) and (ii) feeding activity (area and pattern of bark damage). A gentle soil slope was created around each seedling base to facilitate weevil movement and climbing. The right panel (**b**) conceptual top-view schematic of one experimental cage, illustrating treatment-specific differences in weevil orientation within the cage, showing seedling positions and weevil locations. Coloured plumes schematically indicate qualitative differences in VOC blends emitted by the different treatments. No active airflow or directional air movement was present within the cages

Following VOC exposure, the remaining thirty-two receiver seedlings were transferred to eight experimental cages, with four seedlings placed in each cage—one from each treatment—positioned in the four corners to ensure equal representation of all treatments per cage. Thus, a total of 32 receiver seedlings were used per experimental run, yielding 64 receiver seedlings across both runs. No active airflow was present in Phase 2 cages. Around the base of each seedling, a small mound of soil and peat was shaped into a gentle slope to facilitate weevil access and climbing toward the stem, thereby simulating natural ground conditions (Fig. 2). Seven pine weevils (four males and three females, distinguished by size) were released into the centre of each cage at 08:00, and their distribution among seedlings was recorded at nine time points over the following 24 h (09:00, 10:00, 11:00, 12:00, 15:00, 18:00, 21:00, 23:00, and 08:00 the next morning). Greater emphasis was placed on the early hours of the experiment (09:00–12:00), when weevils were expected to be most active. Because seedling status changed following feeding, early observations provided the clearest measure of weevil responses to VOC exposure before direct feeding altered volatile emissions.

Phase 2 was performed separately for each experimental run using fresh receiver seedlings and a new set of weevils. In total, 16 cages were analysed (*n* = 16 at the cage level, with four seedlings nested per cage). Cages were cleaned and refilled with fresh soil between runs. This temporally staggered setup ensured sufficient time to record observations at all specified time points. To minimize spatial bias, treatment positions were alternated among cages, with different treatments placed in different spatial orientations across experimental units. Bark damage assessments were conducted later, as described in the following sections.

### VOC Sampling

Above-ground parts of 16 exposed receiver seedlings (selected for VOC analysis in Phase 1) were enclosed in pre-conditioned polyethylene terephthalate (PET) cooking bags (30 × 55 cm; LOOK, Fredman Group, Espoo, Finland) to create a dynamic headspace for sampling. In each experimental run, two seedlings per treatment were sampled, totalling four receivers per treatment. The bags were pre-heated at 120 °C for one hour and allowed to return to room temperature before use to minimize contamination from bag materials. Humidified and charcoal-filtered air was supplied from a zero-air generator (Aadco Instruments, 747–30, Cleves, OH, USA) and introduced into each bag. The average inlet flow rate was 600 mL min⁻¹, and the outlet flow rate was 250 mL min⁻¹, creating an overpressure to prevent contamination from ambient air. The inlet and outlet airflows were calibrated using a flow meter (Mini-Buck Calibrator, AP Buck Inc., Orlando, FL, USA). To allow the seedlings to equilibrate to the bag conditions, purified air was flushed through the bags for 30 min. After this adaptation and flushing period, VOCs were collected for 15 min into stainless steel tubes (Markes International Ltd., Llantrisant, UK) filled with Tenax TA and Carbopack B adsorbents (100 mg each, mesh 60/80, Supelco, Bellefonte, PA, USA), connected at the outlet via silicone tubing to a vacuum pump (KNF, D-79112, Germany). Blank samples (empty bags under the same conditions) were also collected in each sampling round to account for background VOC contamination. Immediately after collection, the tubes were sealed with brass caps, refrigerated at + 5 °C, and analysed within 5 days to ensure sample integrity.

### Damage Assessment

**Fig. 3:**
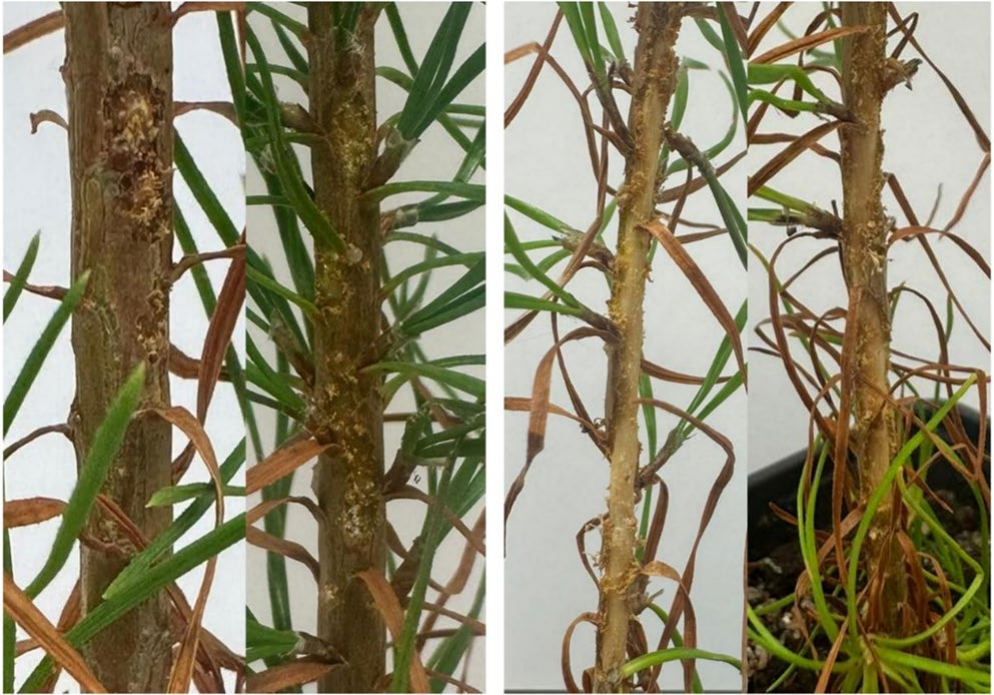
Examples of superficial and deep bark damage caused by pine weevil feeding. The images on the left show superficial damage, limited to the outer bark (periderm), whereas the images on the right show deep damage that extends into the inner bark (phloem)

All receiver seedlings used in the behavioural assay (*n* = 64) were photographed from both sides against a white background with a ruler for scale to standardize the measurements. Photographs were taken after completion of the behavioural experiment. Bark damage was visually classified as either superficial or deep (Fig. 3). Superficial damage was defined as feeding marks limited to the outer bark (periderm), where only the epidermis was affected. Deep damage referred to feeding marks that extended beyond the periderm into the inner bark, visibly affecting a few sub-epidermal cell layers and possibly disrupting the underlying tissues. Damage areas were quantified using ImageJ software (version 1.54 g, Java 1.8.0_345 [64-bit], National Institutes of Health, Bethesda, MD, USA). To better understand weevil feeding behaviour, we used two complementary measures: the total damaged area (cm²) and the number of feeding spots (bite marks) observed on individual seedling bark. While the total damaged area reflects the cumulative tissue removal over a 24-hour continuous feeding period, it does not indicate how often weevils initiated feeding attempts. Counting feeding spots (bite marks) provides complementary information, as deep marks likely represent sustained feeding events, whereas superficial marks reflect exploratory or interrupted feeding. This distinction is important because the number of feeding spots can reveal patterns of host selection and feeding frequency that are not apparent from total damage area alone. Given that weevils were allowed to feed freely for an extended time, the total damaged area mainly reflects the intensity of feeding, whereas the number of spots captures the frequency of feeding attempts.

### VOC Collection, Analysis, and Emission Rate Calculation

VOCs were analysed using a gas chromatography–mass spectrometry (GC–MS) system (GCMS-QP2020, Shimadzu, Kyoto, Japan) equipped with a thermal desorption unit (TD-30R, Shimadzu). Prior to injection, trapped VOCs were thermally desorbed in the TD-30R unit at 300 °C for 10 min and cryofocused at − 20 °C. The cold trap in the TD-30R was packed with the same sorbent materials as described above. The desorbed compounds were transferred in split mode (split ratio 20:1) to a ZB-5MS plus capillary column (60 m × 0.25 mm; film thickness 0.25 μm, Phenomenex Ltd., Torrance, CA, USA) with helium as the carrier gas at a flow rate of 1.88 mL·min⁻¹. The GC oven temperature program started at 40 °C (held for 1 min), increased at 5 °C·min⁻¹ to 125 °C, and then at 10 °C·min⁻¹ to 260 °C, where it was held for 3.5 min. The total run time was 35 min. The mass spectrometer operated in scan mode with an ion source temperature of 200 °C, interface temperature of 280 °C, and a scan range of m/z 33–400. The detector voltage was set at 0.93 kV. Data were analysed using LabSolutions software (Shimadzu). The compounds were identified by comparing their retention times and mass spectra with those in the Wiley library database and with pure standards. For quantification, two customized standard mixtures were used: a terpene standard comprising 21 terpenoid compounds and a green leaf volatile (GLV) standard containing 10 typical green plant volatiles. The detailed composition of these standards is provided in Supplementary Tables 1 and 2. 2 µL of each standard solution was injected into Tenax TA and Carbopack B tubes and analysed alongside the samples to ensure accurate identification and quantification. Compounds not present in the standards were quantified using a representative compound. Selected reference compounds: α-pinene for non-oxygenated monoterpenes (MT-no), 1,8-cineole for oxygenated monoterpenes (MT-ox), trans-caryophyllene for sesquiterpenes (SQTs), and cis-3-hexen-1-ol for other volatile compounds. This grouping followed the method described by Yu et al. (2022). VOC emission rate (*E*, in ng g⁻¹ DM h⁻¹; nanograms per gram of dry mass per hour) was calculated using the following parameters: The mass of VOCs in the sample (Ms) was measured (in nanograms) and normalized to the dry mass of the plant material, while the mass of VOCs in the blank (Mb) was subtracted to correct for background contamination. The flow rate of outgoing air from the sample (*Vs*) and from the blank (*Vb*) were obtained by multiplying the respective airflow rates by the collection time (L·min); the airflow entering the sample (*Fin*) and exiting the sample (*Fout*) were also considered. The dry mass of the above-ground parts of the seedlings (*M*) was measured in grams after oven-drying at 75 °C for 82 h, until constant weight. The collection time (*T*) was set at 0.25 h. Using these parameters, VOC emission rates were calculated with the following equation:

$$\:\boldsymbol{E}=(\mathbf{M}\mathbf{s}\mathbf{}-(\mathbf{M}\mathbf{b}\times\:\boldsymbol{V}\boldsymbol{s}/\boldsymbol{V}\boldsymbol{b})\times\:\boldsymbol{F}\boldsymbol{i}\boldsymbol{n}/\boldsymbol{F}\boldsymbol{o}\boldsymbol{u}\boldsymbol{t})/(\mathbf{M}\times\:\mathbf{T})$$

### Statistical Analyses

All statistical analyses were conducted using IBM SPSS Statistics 29. Differences in weevil distribution among exposure groups over time were analysed using generalized linear mixed models (*GLMMs*), assuming a negative binomial error distribution with a log link function, This approach allows modelling of repeated measurements on the same seedlings over time, incorporates random effects, and is robust to deviations from normality. The number of weevils is the dependent variable, with treatment, time point, and their interaction (treatment × time point) included as fixed effects. Cage identity was included as a random effect to account for repeated observations within cages. Pairwise comparisons were based on estimated marginal means with sequential Bonferroni adjustment. The number of feeding spots (deep and superficial) and the bark damage area (also divided into deep and superficial categories) were analysed to assess two aspects: (1) differences among treatments (W-EXP, S-EXP, M-EXP, and control) and (2) differences between deep and superficial damage within each treatment. The *Kruskal–Wallis* test was used for all between-treatment comparisons, as well as for total bark damage area, as the data were not normally distributed. Within each treatment, deep versus superficial damage was compared using paired *t* tests after confirming normality of the paired differences (deep − superficial) with the *Shapiro–Wilk* test. VOC emission data were first analysed using multivariate analysis of variance (*MANOVA*) to test for overall treatment effects across the multivariate VOC emission profiles, with treatment as the fixed factor and emission rates of individual VOCs included as response variables. Subsequently, and as a separate analysis, one-way *ANOVA* followed by *Tukey’s HSD* post hoc tests were performed for each individual VOC. Prior to conducting parametric tests, the assumptions of normality and homogeneity of variance were verified using *Shapiro–Wilk* and *Levene’s* tests, respectively. Statistical significance was set at *p* ≤ 0.05, with marginal significance considered at *0.05 <* *p ≤ 0.1.*

## Results

### Effects of DIPV Exposure on Weevil Distribution (GLMM Results)

GLMM analysis revealed no significant main effect of treatment (exposure group: Control, M-EXP, W-EXP, and S-EXP; *F* = 0.612, *df* = 3, 540, *p* = 0.608) and no significant main effect of time point (*F* = 1.517, *df* = 8, 540, *p* = 0.148). However, a significant treatment × time point interaction was observed (*F* = 5.755, *df* = 24, 540, *p* < 0.001), indicating that treatment-related differences in weevil attraction varied over time.

**Fig. 4:**
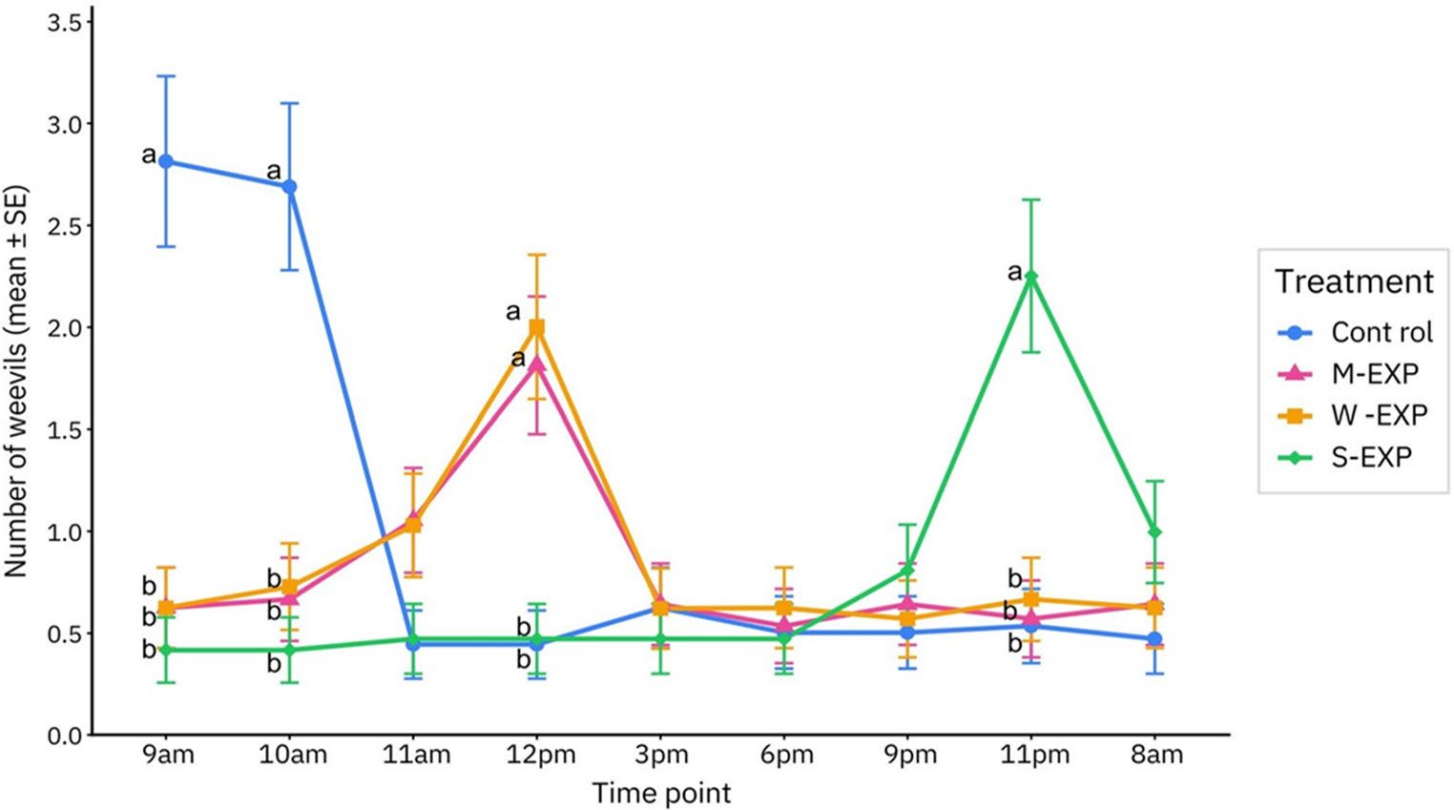
Average number of pine weevils (estimated marginal means ± SE) on receiver Scots pine seedlings exposed to VOCs from control seedlings (Control), mechanically damaged seedlings (M-EXP), weevil-damaged seedlings (W-EXP), or sawfly-damaged seedlings (S-EXP) during a 24-h period. Different letters indicate significant differences between treatments at the same time point based on GLMM pairwise comparisons with sequential Bonferroni adjustment (adjusted *p* < 0.05)

Significant differences among treatments were detected at time points 9 AM, 10 AM, 12 PM, and 11 PM (all adjusted *P* ≤ 0.001), whereas no significant differences were observed at 11 AM, 3 PM, 6 PM, 9 PM, and 8 AM. Pairwise comparisons are reported in Supplementary Table 3. Between 9 and 10 AM, GLMM pairwise comparisons showed that significantly more weevils were observed on control plants than on plants exposed to DIPVs (all adjusted *P* ≤ 0.001). At 12 PM, both M-EXP and W-EXP again had significantly higher weevil counts than Control and S-EXP (all adjusted *P* ≤ 0.001). In S-EXP, significantly more weevils were observed at 11 PM compared with Control, M-EXP, and W-EXP (all adjusted *P* ≤ 0.001) (Fig. 4).

### Bark Damage Areas (cm²) Across Treatment Groups

**Fig. 5:**
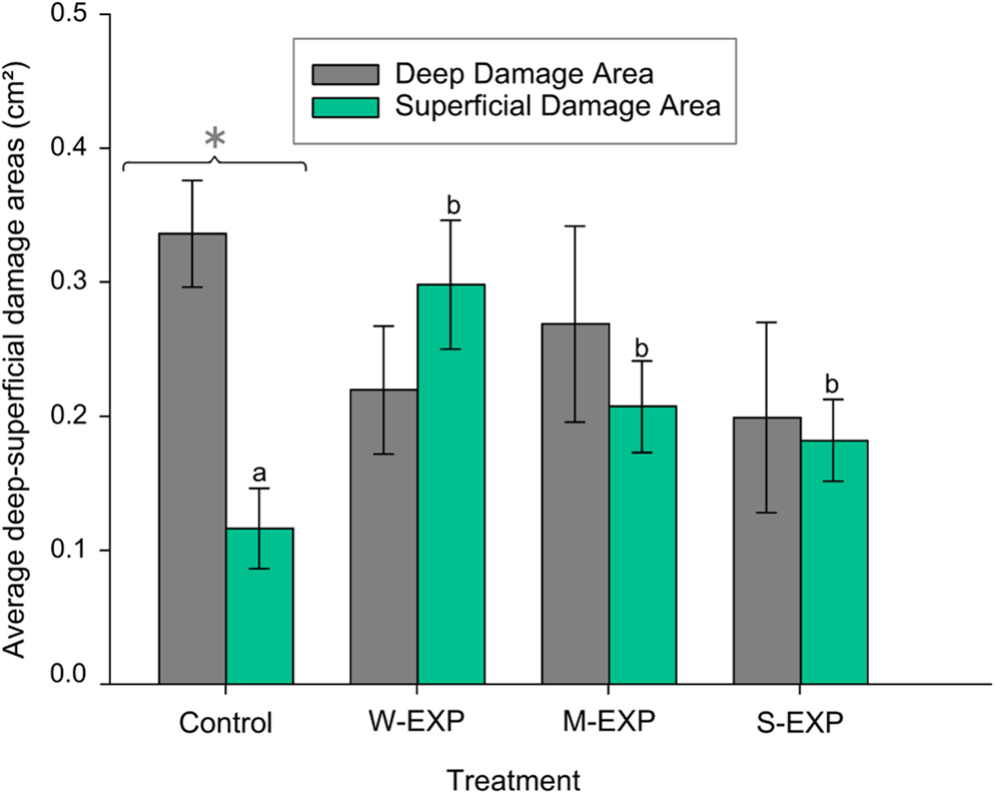
Average deep (grey bars) and superficial (green bars) bark damage areas on Scots pine seedlings exposed to VOCs from control seedlings, mechanically damaged seedlings (M-EXP), weevil-damaged seedlings (W-EXP), and sawfly-damaged seedlings (S-EXP). Error bars represent SE. Different letters indicate significant differences between treatments (*p* < 0.05). The asterisk indicates a significant difference between deep and superficial damage within the control treatment

The extent of bark damage caused by weevil feeding was assessed by measuring the total damaged area (cm²) on each seedling. The total damaged area, including both deep and superficial damage combined, did not differ significantly among treatments. Comparisons within each treatment group showed that in the control group, the deep damage area was greater than the superficial damage area (mean ± SEM: *0.336 ± 0.040* cm² vs. *0.116 ± 0.030* cm²; *P* < 0.001). No significant differences between deep and superficial damage areas were found in the M-EXP and S-EXP groups. In W-EXP, superficial damage was higher on average than deep, but the difference was not statistically significant (0.298 ± 0.048 vs. 0.219 ± 0.048 cm²; *P* = 0.054). Comparisons between treatment groups showed that the control group had a significantly lower superficial damage area than all other exposure groups (*P < 0.010*). Although the deep damage area was highest in the control group, differences from other treatments were not statistically significant (Fig. 5).

### Deep and Superficial Bite Marks as Indicators of Feeding Attempts

**Fig. 6:**
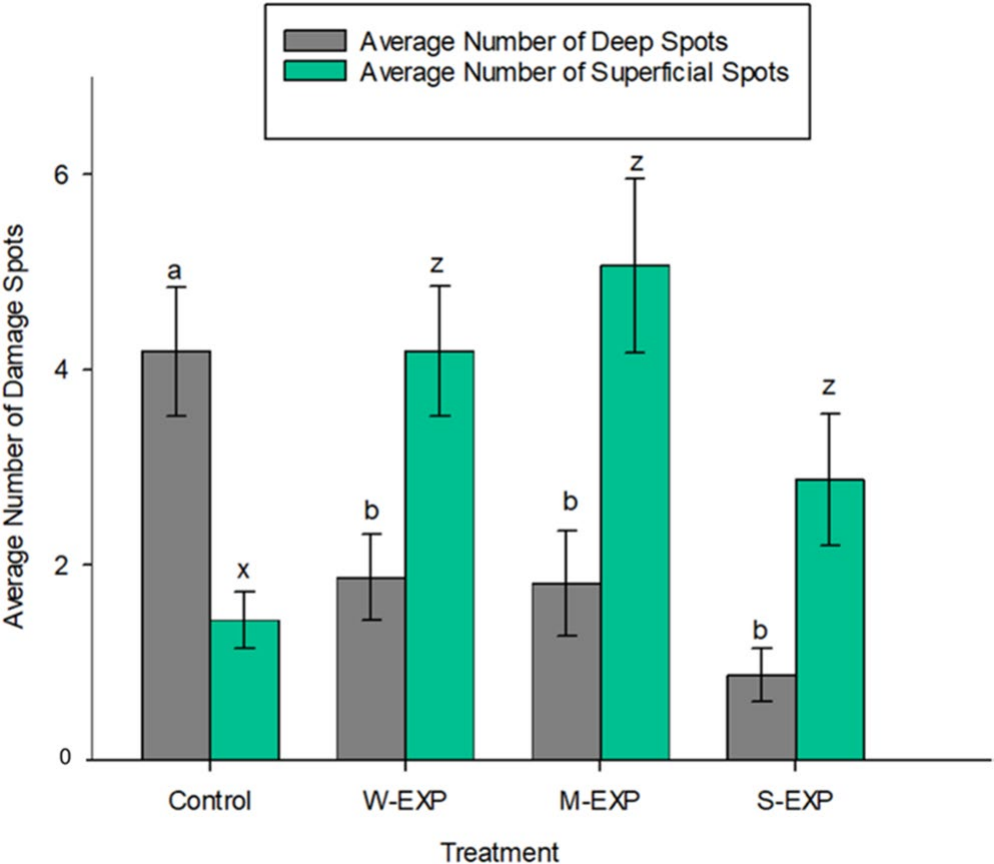
Average number of deep (grey bars) and superficial (green bars) feeding spots on Scots pine seedlings exposed to VOCs from control seedlings, mechanically damaged seedlings (M-EXP), weevil-damaged seedlings (W-EXP), and sawfly-damaged seedlings (S-EXP). Error bars represent SE. Different letters indicate significant differences between treatments (*p* < 0.05); letters (a, b) refer to deep spots, while (x, z) refer to superficial spots

The number of feeding spots, representing visible signs of weevil feeding attempts, was assessed for each seedling (Fig. 6). Within the control group, deep bite marks (4.19 ± 0.660) were significantly more numerous than superficial bite marks (1.44 ± 0.288; *P* < 0.001). Within all exposure groups (M-EXP, W-EXP, and S-EXP), superficial bite marks—regardless of the area of bark removed—were significantly more numerous than deep bite marks (*P < 0.001* for all groups). M-EXP seedlings averaged 1.81 ± 0.542 deep spots and 5.06 ± 0.892 superficial spots, W-EXP seedlings averaged 1.88 ± 0.437 deep spots and 4.19 ± 0.666 superficial spots, and S-EXP seedlings averaged 0.88 ± 0.272 deep spots and 2.88 ± 0.670 superficial spots. Between treatment groups, the control group had significantly more deep bite marks and fewer superficial bite marks than all exposure groups (*P < 0.001*). S-EXP seedlings had significantly fewer deep bite marks than all other treatments (*P < 0.001*).

### Emission Patterns of VOCs in Receiver Seedlings

MANOVA indicated no significant multivariate effect of treatment on the overall VOC emission profile, as assessed by Pillai’s Trace (Pillai’s Trace = 2.596, *F*(36, 9) = 1.607, *p* = 0.23).

**Fig. 7:**
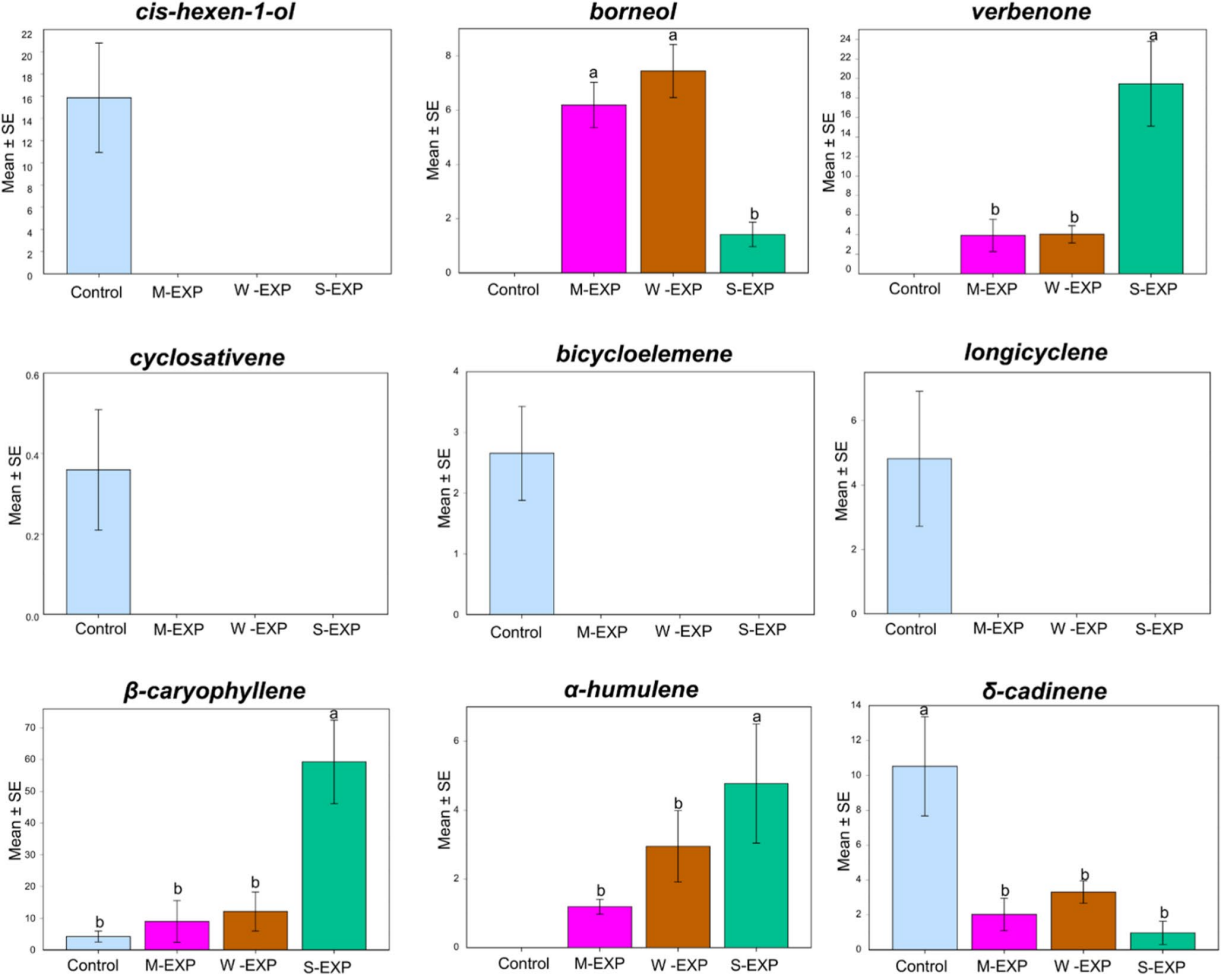
Emission patterns of selected volatile organic compounds (VOCs) with significant differences among exposure groups (control, mechanically damaged [M-EXP], weevil-damaged [W-EXP], and sawfly-damaged [S-EXP]). Values represent mean emission rates (ng g⁻¹ dry matter h⁻¹) ± SE. Different letters above bars indicate significant differences between treatments where applicable, according to Tukey’s HSD test (*p* < 0.05). Compounds showing emissions in only one treatment are presented descriptively without pairwise statistical comparisons

In total, 58 VOCs were emitted by pine receiver seedlings (prior to weevil infestation) after exposure to DIPVs from emitter seedlings. While receiver seedlings exposed to DIPVs from damaged neighbours (W-EXP, S-EXP, and M-EXP) exhibited higher total VOC emissions, including total monoterpenes and both MT-no and MT-ox subgroups, *one-way ANOVA* indicated that these increases were not statistically significant. Among the emitted compounds, nine compounds showed statistically significant differences among treatments based on one-way ANOVA followed by Tukey’s HSD tests (*P* < 0.05). These included two MT-ox compounds, six SQTs, and one compound from the “other VOC” group. Figure 7 illustrates the emission patterns of these nine compounds across the different exposure treatments. Supplementary Table 4 provides the detailed pairwise comparisons for these compounds across exposure groups. Meanwhile, mean (± SE) emission rates for all 58 detected VOCs across treatments are provided in Supplementary Table 5. Notably, verbenone (MT-ox) and β-caryophyllene (SQT) exhibited exceptionally large increases in the sawfly-exposed group compared to other treatments. Additionally, four compounds, including cis-hexen-1-ol, cyclosativene, bicycloelemene, and longicyclene, were detected only in the control group.

## Discussion

Our results demonstrate that exposure to different DIPVs from conspecifics alters host orientation—the process by which large pine weevils select specific seedlings—and feeding behaviour, including the patterns and intensity of their feeding. Pine weevils consistently oriented towards control seedlings, especially during early observation hours, suggesting that DIPVs from emitter seedlings act as cues that reduce the susceptibility of receivers to weevil feeding. Because treatment positions were randomized among experimental units and no directional airflow was present within the cages, this orientation pattern is unlikely to be driven by spatial or directional cues and instead supports a volatile-mediated mechanism underlying weevil host selection. This aligns with previous research indicating that HIPVs can deter herbivores, affect their feeding activity, or prime defences in Scots pines (Yu et al. 2022). Seedlings exposed to VOCs from damaged neighbours showed increased resistance, most clearly reflected in the reduced frequency of deep bark feeding spots. This response could be consistent with priming, whereby prior exposure to DIPVs potentially enhances the subsequent defensive responses against herbivores (Kim and Felton 2013). This is consistent with previous studies showing that HIPVs can enhance defence responses in neighbouring plants by inducing or priming defence-related mechanisms (Engelberth et al. 2004; Yu et al. 2022; Ali et al. 2023).

It is important to note that our experimental system was dynamic, meaning that the seedlings did not remain constant throughout the 24-hour period; their status changed over time, particularly after the initial selection phase when weevils fed on seedlings in each group. For example, the decline in weevil distribution on control seedlings over time may have resulted from VOCs released in response to feeding, which altered the volatile profiles of the seedlings and affected subsequent weevil orientation. These observations supported the concept that plants emit VOCs upon damage, serving as early warning signals that activate defence mechanisms in undamaged tissues (Matsui and Koeduka 2016; Engelberth 2021; Midzi et al. 2022). Given that herbivore feeding in Scots pine is known to induce both local and systemic changes in VOC emissions (Heijari et al. 2011), the behavioural patterns observed here may reflect systemic induction affecting VOC emissions across the entire seedling, rather than a strictly localized effect. In addition, the experimental design provided only a limited set of seedling options, which may have increased the likelihood of weevils selecting less optimal food resources once preferred seedlings had been partially consumed.

Between 3 and 6 p.m., the number of pine weevils observed on seedlings dropped to very low levels. Weevils were present within the cages during this period but were rarely observed on seedlings. This may reflect a decline in feeding motivation after an initial feeding period, leading to reduced abundance on the seedlings. For example, one study showed that providing extra food, such as fresh pine branches near seedlings, can reduce pine weevil damage (Wallertz 2009). This supports the idea that pine weevils often feed intensively at first and then reduce their activity, which may explain the low weevil activity observed during certain hours in our study. Our findings suggest that exposure to DIPVs not only influenced the overall extent of bark damage but also altered the feeding pattern of pine weevils, resulting in a decreased proportion of deep feeding damage and a relative increase in superficial damage. While shifts between deep and superficial feeding damage have not, to our knowledge, been explicitly examined under either field or laboratory conditions, the patterns observed here suggest that such changes may represent a flexible feeding strategy in response to plant condition or induced defences. Although the total damaged surface area did not significantly differ between treatments, the type of damage did: the superficial damage area was significantly lower in the control group compared to the VOC-exposed groups. This indicates that measuring surface area of damage alone may not sufficiently capture qualitative differences in herbivory behaviour. Two seedlings may present similar total damaged surface areas; however, in one the damage may be predominantly superficial, whereas in the other it may involve deeper tissue injury, which is likely to cause greater harm to the plant. As deep feeding is expected to impose higher physiological costs on the plant compared with superficial feeding, such shifts in damage patterns could have important implications for both plant performance and herbivore nutrition. The number of feeding spots reflects the frequency of weevil feeding attempts. In all DIPV-exposed groups, superficial feeding spots were significantly more abundant than deep feeding spots, suggesting either a reduced engagement of weevils in prolonged feeding or the presence of deterrent compounds in DIPV-exposed seedlings that constrain sustained feeding. Although the relative frequency of superficial versus deep feeding has not previously been examined in this context, our findings align with evidence from other plant–insect systems, including conifers, showing that DIPV exposure can activate induced defences and thereby reduce herbivore damage (Keeling and Bohlmann 2006; Pareja and Pinto-Zevallos 2016; Midzi et al. 2022; Niu et al. 2024). In addition, another study in Scots pine demonstrated that HIPV exposure can alter resin duct anatomy, including enlargement of epithelial cell size and the formation of additional epithelial cell layers (Yu et al. 2022). Such structural changes may contribute to the reduced frequency of deep feeding observed in VOC-exposed seedlings.

In our study, weevils exhibited similar behaviour and infestation patterns in seedlings exposed to VOCs from either weevil-damaged or mechanically damaged emitters. This behavioural similarity is consistent with the overall emission patterns observed in receiver seedlings, where mechanically damaged and weevil-damaged treatments showed comparable responses across the compounds that differed significantly among treatments, as well as similar trends for many non-significant compounds reported in Supplementary Tables 4 and 5. Although VOC emissions were measured only in receiver seedlings, the similar pattern in VOC composition between the two treatments indicates that continuous mechanical damage can generate a biologically relevant signal capable of inducing resistance in neighbouring plants, comparable to the HIPVs induced by stem and bark feeding. These findings are consistent with previous studies showing that mechanical damage alone can induce defensive responses and lead to volatile blends similar to those elicited by herbivore attack in species such as *Pinus pinaster* (Moreira et al. 2012), *Phaseolus lunatus* (Mithöfer et al. 2005), and *Cucumis sativus* (Grunseich et al. 2020). Consistently, research on conifers has demonstrated that the type of induction—mechanical or biological—can elicit comparable plant responses, indicating that wounding or methyl jasmonate (MeJA) application may, to some extent, mimic stem-feeding insect attack (McKay et al. 2003; Miller et al. 2005). For example, *P. pinaster* seedlings exhibited similar increases in resin and phenolic compound production following MeJA application, mechanical damage, or weevil feeding (Moreira et al. 2012). Similarly, studies on Scots pine have shown that early stress signalling and defence-related secondary metabolic pathways are induced by mechanical wounding (Lim et al. 2021) as well as by pine weevil herbivory (Kovalchuk et al. 2015). Together, these findings support the interpretation that mechanical damage and weevil feeding can trigger similar olfactory cues in receiver seedlings that influence host recognition. The similarity in VOC profiles and weevil orientation observed here is therefore expected and may partly result from the bark damage applied to emitter seedlings in both treatment groups. However, although no significant differences were found in total damage area or in the number of superficial feeding spots between receivers exposed to mechanically damaged and weevil-damaged emitters, we observed that mechanically induced receivers had a higher deep damage area—ranking second after the control group—and also the greatest number of superficial feeding spots among all groups, indicating frequent feeding attempts. This suggests that VOCs induced by mechanical damage may not induce defences as strongly as VOCs induced by herbivore feeding. Although both groups showed broadly similar patterns and comparable attractiveness to weevils, weevil-induced receivers might be slightly more effective in repelling weevils compared to mechanically induced receivers.

Our findings further demonstrate that defences induced in receivers exposed to VOCs from sawfly feeding made these seedlings less attractive to pine weevils compared with receivers exposed to VOCs induced by bark damage, whether caused by weevil feeding or mechanical wounding, likely reflecting damage-type-specific differences in the DIPV profiles. Previous studies have shown that different herbivores induce different HIPV profiles, leading to variable effects on neighbouring plant resistance across species (Moreira et al. 2012; Joo et al. 2018; Marmolejo et al. 2021). For example, Scots pine exposed to sawfly sex pheromones enhanced its defence against egg deposition (Bittner et al. 2019). While some induced responses extend systemically, most are localized to the damaged tissues (Karban 2011). Weevils showed a specific orientation pattern, visiting sawfly-exposed seedlings later than other groups. This suggests that herbivore identity and the type of damage may contribute to differences in host orientation. Needle-feeding insects, such as sawflies, generally induce volatile blends rich in GLVs and monoterpenes (Ghimire et al. 2013), whereas bark-feeding weevils generate resin-dominated terpenoid profiles with comparatively low GLV levels (Heijari et al. 2011). It is plausible that exposure of receivers to GLV-rich blends—typically associated with needle damage—resulted in volatile profiles that were less familiar or less consistent with host-related cues for pine weevils. Additionally, GLVs are among the earliest chemical signals emitted by plants immediately following herbivore damage (Matsui and Engelberth 2022), a response often termed the ‘GLV burst’ (Mochizuki and Matsui 2018). Even with only indirect exposure to a damaged emitter, such rapid emissions could have shaped volatile cues associated with plant stress or recent damage, which in turn may influence subsequent herbivore interactions. Therefore, the presence of GLV-rich blends in VOCs from sawfly-damaged emitters may represent volatile cues of recent needle damage, which could help explain the altered feeding patterns observed in weevils. On the other hand, bark damage by pine weevils, or even small wounds on seedling stems, can signal a suitable host and attract additional weevils (Tilles et al. 1986; Nordlander 1991; Zagatti et al. 1997). Previous studies indicate that the increased presence of weevils on bark-damaged seedlings is primarily mediated by host plant allelochemicals released from wounds, rather than by aggregation pheromones produced by the weevils themselves (Tilles et al. 1986; Zagatti et al. 1997). This pattern may explain why bark-damage exposure groups were selected earlier than sawfly-induced groups, which were visited latest by weevils.

Although several compounds showed significant differences among exposure groups (Supplementary Table 3), we highlight two that exhibited the greatest variation, particularly in the sawfly-exposed (S-EXP) group. Verbenone, a plant-derived compound known to deter pine weevil feeding (Lindgren et al. 1996; Klepzig and Schlyter 1999), was emitted in significantly higher amounts in this treatment, suggesting that it may function as an effective feeding inhibitor. Another compound, the sesquiterpene β-caryophyllene, also increased significantly in the same treatment, indicating a potential role as a VOC-mediated repellent. While trans-β-caryophyllene has previously been linked to direct weevil damage on Scots pine shoots (Heijari et al. 2011), our results extend these findings by demonstrating that it can also be induced in undamaged receiver seedlings via airborne cues alone. Interestingly, cis-β-caryophyllene emissions were suppressed in Scots pine rhizospheres after shoot herbivory (Rasheed et al. 2021), suggesting stress-related shifts in sesquiterpene regulation. Plants dynamically allocate carbon resources between organs, especially under stress such as herbivore attack (Hartmann et al. 2020). In our experiment, receiver seedlings exposed to DIPVs may have prioritized allocation to the shoots, where defence was most needed, as indicated by an increase in β-caryophyllene in the shoots of exposed seedlings. This pattern may indicate a stress-induced shift in carbon allocation, with more resources directed to the biosynthesis of defence compounds in aboveground tissues, including β-caryophyllene. Although the emission observed here was induced in undamaged receiver seedlings and restricted to aboveground tissues, the fact that pine weevils were visited last in the S-EXP treatment supports a broader defensive role for β-caryophyllene beyond localized, damage-dependent responses. This interpretation is consistent with studies identifying β-caryophyllene as an insect-repelling sesquiterpene across diverse ecological systems (Liu et al. 2010). Additional support for the repellent function of β-caryophyllene comes from studies on other weevil species within the family Curculionidae. For example, raspberry weevils were deterred by essential oils composed almost exclusively of sesquiterpenes (99.5%) (Espinoza et al. 2016), and β-caryophyllene reduced feeding and reproduction in both rice and maize weevils (Chaubey 2012, 2022). Although these species feed on different plants, they all belong to the same weevil family. This suggests that β-caryophyllene may be an active defence compound produced by Scots pine when attacked or stressed and may also have a general repellent effect across various weevil species within the same family.

## Conclusion

This study provides new insight into how pre-exposure of Scots pine seedlings to DIPVs reshapes pine weevil orientation and feeding behaviour. Receivers exposed to damaged neighbour cues experienced less deep bark removal and more superficial feeding spots, indicating enhanced resistance. Repeated mechanical injury produced receiver VOC profiles and behavioural outcomes comparable to those elicited by weevil feeding, whereas sawfly-induced blends were associated with the latest attraction. The composition of DIPVs, shaped by herbivore identity and damage type, emerged as a key driver of host selection and feeding patterns. By integrating behavioural observations, damage assessments, and chemical analyses, our findings underscore the potential ecological importance of damage-induced plant–plant signalling in mediating plant-herbivore interactions in coniferous forests. Further research under natural field conditions is needed to explore the persistence and ecological benefits of VOC-mediated resistance, as well as its broader implications for forest resilience.

## Supplementary Information

Below is the link to the electronic supplementary material.

Supplementary Material 1 (DOCX 116 KB)

Supplementary Material 2 (DOCX 37.6 KB)

Supplementary Material 3 (DOCX 22.3 KB)

Supplementary Material 4 (DOCX 39.6 KB)

## Data Availability

The data used to support the findings of this study are available from the corresponding author upon request.
