# Supplementary material for: Effects of Exposure to Differentially Stressed Pinus sylvestris Seedlings on the Susceptibility of Receivers to Feeding by the Large Pine Weevil
Source: J Chem Ecol. 2026 Feb 21;52(2):21. doi: 10.1007/s10886-026-01688-5 (PMC12923392; doi:10.1007/s10886-026-01688-5)
Supplement: Supplementary file 1 — Supplementary Material 1 (DOCX 116 KB) [file 10886_2026_1688_MOESM1_ESM.docx]

**Supplementary Table 1**

| Compounds (21 terpene) |
| --- |
| α-Pinene (>97 %) |
| Camphene (95 %) |
| Sabinene (70 %) |
| β-Pinene (99 %) |
| β-Myrcene (~90 %) |
| 3-Carene (95 %) |
| D-Limonene |
| 1,8-Cineole (~99 %) |
| γ-Terpinene (95 %) |
| Terpinolene (~90 %) |
| Linalool (~97 %) |
| (*E*)-DMNT |
| Camphor (96 %) |
| Borneol (98 %) |
| Terpinen-4-ol (97 %) |
| α-Terpineol (98 %) |
| Bornylacetate (~97 %) |
| Longifolene (>99 %) |
| (*E*)-β-Farnesene |
| α-Humulene (>98 %) |
| α-Copaene (~95 %) |
| (*E*)-Caryophyllene |
| Aromadendrene (>97 %) |
| β-Elemene |
| α-Phellandrene (>95 %) |

**Table 1.** Supplementary Table 1. List of 21 terpene standards used for GC–MS compound identification. Percentage purities are provided where available.

**Supplementary Table 2**

| Compounds (GLV) |
| --- |
| cis-3-hexen-1-ol (98%) |
| trans-2-hexenal (98%) |
| 1-hexanol (98%) |
| 1-octen-3-ol (>97%) |
| cis-3-hexenyl acetate (98%) |
| nonanal (95%) |
| cis-3-hexenyl butyrate (98%) |
| methyl salicylate (99%) |
| cis-3-hexenyl isovalerate |
| cis-3-hexenyl tiglate (97%) |

**Table 2.** List of GLV standards used for compound identification. Percentage purities are provided where available.
