# Supplementary material for: Effects of Exposure to Differentially Stressed Pinus sylvestris Seedlings on the Susceptibility of Receivers to Feeding by the Large Pine Weevil
Source: J Chem Ecol. 2026 Feb 21;52(2):21. doi: 10.1007/s10886-026-01688-5 (PMC12923392; doi:10.1007/s10886-026-01688-5)
Supplement: Supplementary file 2 — Supplementary Material 2 (DOCX 37.6 KB) [file 10886_2026_1688_MOESM2_ESM.docx]

**Supplementary Table 3**

| TimePoint | Treatment Pairwise Contrasts | Contrast Estimate | Std. Error | t | Adj. Sig. | 95% Confidence Interval | |
| --- | --- | --- | --- | --- | --- | --- | --- |
|  |  |  |  |  |  | Lower | Upper |
| 9 AM | Control - Mechanical | 2.190 | .464 | 4.724 | <.001 | .992 | 3.388 |
|  | Control - Weevil | 2.190 | .464 | 4.724 | <.001 | .992 | 3.388 |
|  | Control - Sawfly | 2.397 | .449 | 5.335 | <.001 | 1.207 | 3.587 |
|  | Mechanical - Control | -2.190 | .464 | -4.724 | <.001 | -3.388 | -.992 |
|  | Mechanical - Weevil | <.001 | .279 | <.001 | 1.000 | -.549 | .549 |
|  | Mechanical - Sawfly | .207 | .255 | .813 | 1.000 | -.384 | .799 |
|  | Weevil - Control | -2.190 | .464 | -4.724 | <.001 | -3.388 | -.992 |
|  | Weevil - Mechanical | <.001 | .279 | <.001 | 1.000 | -.549 | .549 |
|  | Weevil - Sawfly | .207 | .255 | .813 | 1.000 | -.405 | .820 |
|  | Sawfly - Control | -2.397 | .449 | -5.335 | <.001 | -3.587 | -1.207 |
|  | Sawfly - Mechanical | -.207 | .255 | -.813 | 1.000 | -.799 | .384 |
|  | Sawfly - Weevil | -.207 | .255 | -.813 | 1.000 | -.820 | .405 |
| 10 AM | Control - Mechanical | 2.024 | .458 | 4.419 | <.001 | .840 | 3.208 |
|  | Control - Weevil | 1.962 | .462 | 4.245 | <.001 | .804 | 3.120 |
|  | Control - Sawfly | 2.273 | .441 | 5.159 | <.001 | 1.106 | 3.440 |
|  | Mechanical - Control | -2.024 | .458 | -4.419 | <.001 | -3.208 | -.840 |
|  | Mechanical - Weevil | -.062 | .295 | -.210 | .834 | -.642 | .518 |
|  | Mechanical - Sawfly | .249 | .260 | .958 | .735 | -.344 | .842 |
|  | Weevil - Control | -1.962 | .462 | -4.245 | .000 | -3.120 | -.804 |
|  | Weevil - Mechanical | .062 | .295 | .210 | .834 | -.518 | .642 |
|  | Weevil - Sawfly | .311 | .268 | 1.164 | .735 | -.331 | .954 |
|  | Sawfly - Control | -2.273 | .441 | -5.159 | <.001 | -3.440 | -1.106 |
|  | Sawfly - Mechanical | -.249 | .260 | -.958 | .735 | -.842 | .344 |
|  | Sawfly - Weevil | -.311 | .268 | -1.164 | .735 | -.954 | .331 |
| 11 AM | Control - Mechanical | -.610 | .306 | -1.995 | .279 | -1.421 | .200 |
|  | Control - Weevil | -.585 | .303 | -1.929 | .279 | -1.372 | .202 |
|  | Control - Sawfly | -.029 | .239 | -.120 | 1.000 | -.566 | .509 |
|  | Mechanical - Control | .610 | .306 | 1.995 | .279 | -.200 | 1.421 |
|  | Mechanical - Weevil | .025 | .361 | .070 | 1.000 | -.692 | .743 |
|  | Mechanical - Sawfly | .582 | .309 | 1.883 | .279 | -.209 | 1.372 |
|  | Weevil - Control | .585 | .303 | 1.929 | .279 | -.202 | 1.372 |
|  | Weevil - Mechanical | -.025 | .361 | -.070 | 1.000 | -.743 | .692 |
|  | Weevil - Sawfly | .556 | .306 | 1.817 | .279 | -.211 | 1.324 |
|  | Sawfly - Control | .029 | .239 | .120 | 1.000 | -.509 | .566 |
|  | Sawfly - Mechanical | -.582 | .309 | -1.883 | .279 | -1.372 | .209 |
|  | Sawfly - Weevil | -.556 | .306 | -1.817 | .279 | -1.324 | .211 |
| 12 PM | Control - Mechanical | -1.371 | .376 | -3.648 | .001 | -2.312 | -.429 |
|  | Control - Weevil | -1.559 | .391 | -3.986 | .000 | -2.594 | -.523 |
|  | Control - Sawfly | -.029 | .239 | -.120 | 1.000 | -.509 | .452 |
|  | Mechanical - Control | 1.371 | .376 | 3.648 | .001 | .429 | 2.312 |
|  | Mechanical - Weevil | -.188 | .488 | -.385 | 1.000 | -1.286 | .910 |
|  | Mechanical - Sawfly | 1.342 | .378 | 3.550 | .001 | .434 | 2.250 |
|  | Weevil - Control | 1.559 | .391 | 3.986 | .000 | .523 | 2.594 |
|  | Weevil - Mechanical | .188 | .488 | .385 | 1.000 | -.910 | 1.286 |
|  | Weevil - Sawfly | 1.530 | .393 | 3.890 | .001 | .513 | 2.547 |
|  | Sawfly - Control | .029 | .239 | .120 | 1.000 | -.452 | .509 |
|  | Sawfly - Mechanical | -1.342 | .378 | -3.550 | .001 | -2.250 | -.434 |
|  | Sawfly - Weevil | -1.530 | .393 | -3.890 | .001 | -2.547 | -.513 |
| 3 PM | Control - Mechanical | -.018 | .281 | -.064 | 1.000 | -.577 | .541 |
|  | Control - Weevil | .003 | .279 | .012 | 1.000 | -.546 | .552 |
|  | Control - Sawfly | .152 | .262 | .580 | 1.000 | -.425 | .728 |
|  | Mechanical - Control | .018 | .281 | .064 | 1.000 | -.541 | .577 |
|  | Mechanical - Weevil | .021 | .281 | .075 | 1.000 | -.538 | .581 |
|  | Mechanical - Sawfly | .170 | .264 | .643 | 1.000 | -.529 | .869 |
|  | Weevil - Control | -.003 | .279 | -.012 | 1.000 | -.552 | .546 |
|  | Weevil - Mechanical | -.021 | .281 | -.075 | 1.000 | -.581 | .538 |
|  | Weevil - Sawfly | .148 | .261 | .568 | 1.000 | -.426 | .723 |
|  | Sawfly - Control | -.152 | .262 | -.580 | 1.000 | -.728 | .425 |
|  | Sawfly - Mechanical | -.170 | .264 | -.643 | 1.000 | -.869 | .529 |
|  | Sawfly - Weevil | -.148 | .261 | -.568 | 1.000 | -.723 | .426 |
| 6 PM | Control - Mechanical | -.032 | .255 | -.127 | 1.000 | -.544 | .480 |
|  | Control - Weevil | -.121 | .265 | -.457 | 1.000 | -.691 | .448 |
|  | Control - Sawfly | .030 | .247 | .123 | 1.000 | -.465 | .526 |
|  | Mechanical - Control | .032 | .255 | .127 | 1.000 | -.480 | .544 |
|  | Mechanical - Weevil | -.089 | .269 | -.330 | 1.000 | -.652 | .474 |
|  | Mechanical - Sawfly | .063 | .251 | .251 | 1.000 | -.453 | .579 |
|  | Weevil - Control | .121 | .265 | .457 | 1.000 | -.448 | .691 |
|  | Weevil - Mechanical | .089 | .269 | .330 | 1.000 | -.474 | .652 |
|  | Weevil - Sawfly | .152 | .262 | .580 | 1.000 | -.541 | .845 |
|  | Sawfly - Control | -.030 | .247 | -.123 | 1.000 | -.526 | .465 |
|  | Sawfly - Mechanical | -.063 | .251 | -.251 | 1.000 | -.579 | .453 |
|  | Sawfly - Weevil | -.152 | .262 | -.580 | 1.000 | -.845 | .541 |
| 9 PM | Control - Mechanical | -.139 | .267 | -.521 | 1.000 | -.721 | .442 |
|  | Control - Weevil | -.067 | .259 | -.259 | 1.000 | -.601 | .467 |
|  | Control - Sawfly | -.305 | .286 | -1.065 | 1.000 | -1.063 | .453 |
|  | Mechanical - Control | .139 | .267 | .521 | 1.000 | -.442 | .721 |
|  | Mechanical - Weevil | .072 | .275 | .263 | 1.000 | -.495 | .640 |
|  | Mechanical - Sawfly | -.166 | .301 | -.550 | 1.000 | -.824 | .493 |
|  | Weevil - Control | .067 | .259 | .259 | 1.000 | -.467 | .601 |
|  | Weevil - Mechanical | -.072 | .275 | -.263 | 1.000 | -.640 | .495 |
|  | Weevil - Sawfly | -.238 | .293 | -.811 | 1.000 | -.918 | .442 |
|  | Sawfly - Control | .305 | .286 | 1.065 | 1.000 | -.453 | 1.063 |
|  | Sawfly - Mechanical | .166 | .301 | .550 | 1.000 | -.493 | .824 |
|  | Sawfly - Weevil | .238 | .293 | .811 | 1.000 | -.442 | .918 |
| 11 PM | Control - Mechanical | -.035 | .263 | -.131 | 1.000 | -.563 | .494 |
|  | Control - Weevil | -.131 | .274 | -.478 | 1.000 | -.789 | .527 |
|  | Control - Sawfly | -1.716 | .417 | -4.112 | .000 | -2.821 | -.611 |
|  | Mechanical - Control | .035 | .263 | .131 | 1.000 | -.494 | .563 |
|  | Mechanical - Weevil | -.096 | .278 | -.347 | 1.000 | -.679 | .486 |
|  | Mechanical - Sawfly | -1.681 | .420 | -4.004 | <.001 | -2.767 | -.596 |
|  | Weevil - Control | .131 | .274 | .478 | 1.000 | -.527 | .789 |
|  | Weevil - Mechanical | .096 | .278 | .347 | 1.000 | -.486 | .679 |
|  | Weevil - Sawfly | -1.585 | .427 | -3.712 | <.001 | -2.655 | -.515 |
|  | Sawfly - Control | 1.716 | .417 | 4.112 | <.001 | .611 | 2.821 |
|  | Sawfly - Mechanical | 1.681 | .420 | 4.004 | <.001 | .596 | 2.767 |
|  | Sawfly - Weevil | 1.585 | .427 | 3.712 | .001 | .515 | 2.655 |
| 8 AM | Control - Mechanical | -.170 | .264 | -.643 | 1.000 | -.759 | .419 |
|  | Control - Weevil | -.152 | .262 | -.580 | 1.000 | -.728 | .425 |
|  | Control - Sawfly | -.524 | .303 | -1.730 | .506 | -1.326 | .278 |
|  | Mechanical - Control | .170 | .264 | .643 | 1.000 | -.419 | .759 |
|  | Mechanical - Weevil | .018 | .281 | .064 | 1.000 | -.541 | .577 |
|  | Mechanical - Sawfly | -.354 | .320 | -1.107 | 1.000 | -1.148 | .439 |
|  | Weevil - Control | .152 | .262 | .580 | 1.000 | -.425 | .728 |
|  | Weevil - Mechanical | -.018 | .281 | -.064 | 1.000 | -.577 | .541 |
|  | Weevil - Sawfly | -.372 | .318 | -1.170 | 1.000 | -1.195 | .450 |
|  | Sawfly - Control | .524 | .303 | 1.730 | .506 | -.278 | 1.326 |
|  | Sawfly - Mechanical | .354 | .320 | 1.107 | 1.000 | -.439 | 1.148 |
|  | Sawfly - Weevil | .372 | .318 | 1.170 | 1.000 | -.450 | 1.195 |

**Panel A.** Results of pairwise comparisons among treatments (Control, Mechanical-EXP, Weevil-EXP, and Sawfly-EXP) at each time point based on generalized linear mixed-effects models (GLMMs). The table reports estimated contrasts between treatments, including both statistically significant and non-significant comparisons. Contrast estimates represent differences between estimated marginal means of treatments. Standard errors refer to the contrast estimates (not raw means). Adjusted p-values and 95% confidence intervals are provided.

| **Overall Test Results** | | | | |
| --- | --- | --- | --- | --- |
| TimePoint | F | df1 | df2 | Sig. |
| 9 AM | 9.568 | 3 | 540 | <.001 |
| 10 AM | 8.890 | 3 | 540 | <.001 |
| 11 AM | 2.431 | 3 | 540 | .064 |
| 12 PM | 9.501 | 3 | 540 | <.001 |
| 3 PM | .194 | 3 | 540 | .901 |
| 6 PM | .121 | 3 | 540 | .948 |
| 9 PM | .403 | 3 | 540 | .751 |
| 11 PM | 6.129 | 3 | 540 | <.001 |
| 8 AM | .999 | 3 | 540 | .393 |

**Panel B.** Overall test results for treatment effects at each time point based on generalized linear mixed-effects models (GLMMs).
