# Supplementary material for: Effects of Exposure to Differentially Stressed Pinus sylvestris Seedlings on the Susceptibility of Receivers to Feeding by the Large Pine Weevil
Source: J Chem Ecol. 2026 Feb 21;52(2):21. doi: 10.1007/s10886-026-01688-5 (PMC12923392; doi:10.1007/s10886-026-01688-5)
Supplement: Supplementary file 3 — Supplementary Material 3 (DOCX 22.3 KB) [file 10886_2026_1688_MOESM3_ESM.docx]

**Supplementary Table 4**

| Compound group | Compound | Treatment | mean of emission (ng g^−1^ DM h^−1^) | Standard Error of Mean (SEM) | Significant Pairwise Comparisons (Tukey HSD, p < 0.05) |
| --- | --- | --- | --- | --- | --- |
| **Other** | **Cis-Hexen-1-ol** | Control | 15.865 | 4.9321 | * |
|  |  | Mechanical | 0 | 0 | Emission detected only in control |
|  |  | Weevil | 0 | 0 |  |
|  |  | Sawfly | 0 | 0 |  |
| **Monoterpenes** | **Borneol** | Control | 0 | 0 | (zero emission) |
|  |  | Mechanical | 6.19 | 0.83835 | b |
|  |  | Weevil | 7.43 | 0.9547 |  |
|  |  | Sawfly | 1.42 | 0.45065 | a |
|  | **Verbenone** | Control | 0 | 0 | (zero emission) |
|  |  | Mechanical | 3.91 | 1.6521 | b |
|  |  | Weevil | 4.03 | 0.8867 |  |
|  |  | Sawfly | 19.45 | 4.34775 | a |
| **Sesquiterpenes** | **Cyclosativene** | Control | 0.3594 | 0.1492 | * |
|  |  | Mechanical | 0 | 0 | Emission detected only in control |
|  |  | Weevil | 0 | 0 |  |
|  |  | Sawfly | 0 | 0 |  |
|  | **Bicycloelemene** | Control | 2.6549 | 0.7759 | * |
|  |  | Mechanical | 0 | 0 | Emission detected only in control |
|  |  | Weevil | 0 | 0 |  |
|  |  | Sawfly | 0 | 0 |  |
|  | **Longicyclene** | Control | 4.821 | 2.09915 | * |
|  |  | Mechanical | 0 | 0 | Emission detected only in control |
|  |  | Weevil | 0 | 0 |  |
|  |  | Sawfly | 0 | 0 |  |
|  | **β-caryophyllene** | Control | 4.2028 | 1.74365 | b |
|  |  | Mechanical | 8.9663 | 6.5867 |  |
|  |  | Weevil | 12.1193 | 6.1576 |  |
|  |  | Sawfly | 59.3157 | 13.1391 | a |
|  | **α-humulene** | Control | 0 | 0 | (zero emission) |
|  |  | Mechanical | 1.193 | 0.2153 | a |
|  |  | Weevil | 2.947 | 1.0393 |  |
|  |  | Sawfly | 4.773 | 1.7304 | b |
|  | **δ-Cadinene** | Control | 10.5203 | 2.843 | a |
|  |  | Mechanical | 2.0272 | 0.9259 | b |
|  |  | Weevil | 3.3109 | 0.6442 |  |
|  |  | Sawfly | 0.9679 | 0.6626 |  |

**Table 4.** Emissions of selected (VOCs), including monoterpenes, sesquiterpenes, and other compounds, showing significant differences among exposure groups (control, mechanically damaged [M-EXP], weevil-damaged [W-EXP], and sawfly-damaged [S-EXP]). Values are mean emission rates (ng g⁻¹ dry mass h⁻¹) ± standard error of the mean (SEM). Significant pairwise differences between treatments were determined using Tukey’s HSD test (p < 0.05).
