# Supplementary material for: Effects of Exposure to Differentially Stressed Pinus sylvestris Seedlings on the Susceptibility of Receivers to Feeding by the Large Pine Weevil
Source: J Chem Ecol. 2026 Feb 21;52(2):21. doi: 10.1007/s10886-026-01688-5 (PMC12923392; doi:10.1007/s10886-026-01688-5)
Supplement: Supplementary file 4 — Supplementary Material 4 (DOCX 39.6 KB) [file 10886_2026_1688_MOESM4_ESM.docx]

**Supplementary Table 5**

| **Compound** | **Control**  **(mean ± SE)** | **M-EXP**  **(mean ± SE)** | **W-EXP**  **(mean ± SE)** | **S-EXP**  **(mean ± SE)** | **ANOVA Sig.**  **Between Groups** |
| --- | --- | --- | --- | --- | --- |
| Unknown1 | 20.53±8.65 | 28.98±9.94 | 26.71±9.36 | 22.87±5.31 | .896 |
| **cis-hexen-1-ol** | 15.86±4.93 | 0 | 0 | 0 | .001 |
| Heptanal | 3.50±2.06 | 4.07±1.91 | 1.15±1.1 | 6.79±3.39 | .414 |
| Tricyclene | 16.55±11.5 | 4.32±1.83 | 18.28±8.87 | 7.41±5.38 | .538 |
| α-Thujene | 3.53±2.85 | 1.89±0.76 | 2.86±1.07 | 1.22±1.17 | .774 |
| α-Pinene | 7,678.75±5,711.6 | 1,749.50±1,360.1 | 3,803.1±422.1 | 1,116.6±741.2 | .428 |
| α-Fenchene | 3.23±2.72 | 0.84±0.8 | 4.67±3.18 | 0 | .421 |
| Camphene | 167.50±124.4 | 40.05±20.89 | 134.69±42.71 | 57.03±39.29 | .531 |
| Berbenone | 10.11±8.87 | 1.70±1.7 | 4.88±2.56 | 0.48±0.29 | .499 |
| Sabinene | 26.52±20.18 | 10.51±10.5 | 12.41±8.99 | 12.32±12.3 | .828 |
| β-Pinene | 525.92±409.38 | 193.79±122.20 | 224.17±38.87 | 122.78±91.18 | .593 |
| Myrcene | 959.41±878.22 | 242.88±167.19 | 123.70±29.75 | 485.51±471.63 | .667 |
| cis-3-hexenyl acetate | 1.81±1.26 | 0 | 0 | 0 | .160 |
| α-Phellandrene | 13.74±12.47 | 2.14±0.89 | 3.90±1.38 | 3.09±2.85 | .570 |
| Δ³-Carene | 21.27±11.67 | 51.0±27.73 | 30.77±17.64 | 6.50±2.60 | .374 |
| α-Terpinene | 6.38±4.63 | 2.53±1.13 | 2.88±0.49 | 1.65±1.6 | .586 |
| p-Cymene | 10.69±8.24 | 6.44±3.75 | 11.27±5.17 | 3.10±3.1 | .690 |
| Limonene | 329.45±196.31 | 260.49±181.42 | 431.22±342.90 | 540.79±467.90 | .930 |
| β-Phellandrene | 402.97±368.26 | 67.23±30.92 | 105.24±48.96 | 181.68±154.14 | .655 |
| 1,8-Cineole | 0 | 0 | 1.96±1.20 | 0 | .097 |
| trans-β-Ocimene | 19.40±10.70 | 9.15±4.39 | 87.52±77.45 | 7.40±2.88 | .449 |
| γ-Terpinene | 10.85±7.11 | 5.66±2.21 | 4.36±0.70 | 2.89±1.76 | .512 |
| Terpinolene | 42.08±35.04 | 24.52±14.60 | 13.63±3.70 | 6.86±6.0 | .608 |
| Methylphenyl propanal | 1.59±0.93 | 2.06±1.27 | 5.20±3.20 | 0.86±0.53 | .379 |
| Linalool | 11.49±10.67 | 3.06±1.83 | 0.42±0.4 | 0.41±0.4 | .454 |
| Nonanal | 32.70±22.34 | 19.10±11.97 | 13.46±6.89 | 46.88±28.40 | .630 |
| Allo-Ocimene | 4.77±1.53 | 6.08±1.31 | 4.45±1.60 | 6.01±1.87 | .840 |
| Camphor | 2.18±0.87 | 2.13±1.2 | 3.15±0.79 | 3.51±1.84 | .824 |
| Pinocarvone | 14.14±13.35 | 1.41±1.4 | 1.94±0.58 | 0.94±0.72 | .472 |
| **Borneol** | 0 | 6.18±0.83 | 7.43±0.95 | 1.41±0.45 | <.001 |
| Terpinen-4-ol | 0.91±0.58 | 0 | 0.08±0.08 | 0.61±0.6 | .408 |
| α-Terpineol | 1.38±1.3 | 0 | 0 | 0.39±0.3 | .505 |
| Myrtenal | 9.92±9.4 | 1.23±1.2 | 0.91±0.53 | 0 | .452 |
| **Verbenone** | 0 | 3.91±1.65 | 4.02±0.88 | 19.45±4.34 | <.001 |
| Bornyl acetate | 31.75±29.45 | 3.04±1.76 | 6.89±3.44 | 22.86±20.54 | .653 |
| Unknown2 | 1.19±0.45 | 2.32±0.81 | 1.70±0.58 | 1.62±0.6 | .661 |
| **Bicycloelemene** | 2.65±0.77 | 0 | 0 | 0 | <.001 |
| α-Terpinyl acetate | 2.03±1.25 | 0.65±0.37 | 0.37±0.26 | 0 | .209 |
| **Cyclosativene** | 0.35±0.14 | 0 | 0 | 0 | <.001 |
| α-Copaene | 0.47±0.4 | 0.26±0.2 | 1.28±0.68 | 0.34±0.3 | .423 |
| **Longicyclene** | 4.82±2.09 | 0 | 0 | 0 | .015 |
| β-Elemene | 9.52±5.75 | 1.59±1.5 | 2.87±1.17 | 2.03±2.0 | .307 |
| Longifolene | 23.48±21.5 | 0.14±0.1 | 2.25±1.02 | 0 | .382 |
| **β-caryophyllene** | 4.20±1.74 | 8.96±6.58 | 12.11±6.15 | 59.31±13.13 | .001 |
| β-Cubebene | 0.89±0.8 | 0.65±0.6 | 0.86±0.5 | 0 | .729 |
| Geranyl acetone | 2.16±1.47 | 3.28±2.04 | 2.74±2.7 | 2.49±1.74 | .983 |
| Aromadendrene | 1.85±0.76 | 0 | 1.85±1.8 | 0 | .371 |
| **α-humulene** | 0 | 1.19±0.21 | 2.94±1.03 | 4.77±1.73 | .030 |
| γ-Cadinene | 1.27±0.52 | 0.57±0.33 | 0.36±0.22 | 0.55±0.5 | .488 |
| Azulene | 1.14±0.68 | 0 | 0.14±0.1 | 0 | .108 |
| D-Germacrene | 2.37±1.36 | 2.56±1.69 | 0.36±0.3 | 1.036±1.03 | .540 |
| α-Amorphene | 1.30±1.3 | 0 | 1.3±0.75 | 0 | .434 |
| α-Muurolene | 2.67±1.86 | 1.06±0.62 | 1.76±0.75 | 0.71±0.33 | .592 |
| Bicyclogermacrene | 18.80±13.25 | 1.89±1.5 | 4.08±1.60 | 3.25±3.25 | .317 |
| **δ-Cadinene** | 10.52±2.84 | 2.027±0.92 | 3.31±0.64 | 0.92±0.66 | .004 |
| cis-Bisabolene | 0.58±0.35 | 0.37±0.3 | 0.45±0.4 | 0.10±0.1 | .788 |
| α-Cadinene | 0.47±0.36 | 0 | 0 | 0 | .235 |
| α-Bisabolol | 1.60±1.30 | 1.81±1.04 | 0 | 3.36±1.94 | .369 |

**Table 5.** Mean (± SE) VOC emission rates for control, mechanically damaged (M-EXP), weevil-damaged (W-EXP), and sawfly-damaged (S-EXP) emitter seedlings. Values are mean emission rates (ng g⁻¹ dry matter h⁻¹). Compounds showing significant differences among treatments based on one-way ANOVA (p < 0.05) are highlighted in bold and shaded in blue.
